# Golden leaf formation of *Populus nigra* is associated with the up-regulation of *Stay-Green* expression

**DOI:** 10.1186/s43897-025-00204-9

**Published:** 2026-03-06

**Authors:** Wanting Fu, Zimeng Li, Xiaoou Zhai, Haizhen Zhang, Shuang Feng, Aimin Zhou

**Affiliations:** 1 College of Horticulture and Landscape Architecture, Northeast Agricultural University, Harbin, 150030 China; 2 Heilongjiang Forest Botanical Garden, Harbin, 150046 China; 3 Large-Scale Instrument and Equipment Sharing Service Platform, Northeast Agricultural University, Harbin, 150030 China

*Populus nigra* is an important garden and ecological tree species valued for its rapid growth and distinctive morphology. The golden leaf cultivar *P. nigra* cv. “Jinye” (JY) is a naturally occurring variant with considerable ornamental and economic significance. However, the molecular basis underlying the formation of “JY” remains unclear. Unraveling the mechanisms responsible for golden leaf development in “JY” is therefore essential for advancing colored-leaf breeding in poplar and other ornamental woody species.

Chlorophyll (Chl) is the primary determinant of leaf coloration and a key component of photosynthesis (Wang et al. 2020). In higher plants, Chl metabolism involves three major processes: biosynthesis, the Chl cycle (interconversion of Chl a and Chl b), and degradation (Mochizuki et al. 2010; Woo et al. 2019). Suppression of Chl biosynthesis or acceleration of Chl degradation leads to reduced Chl content, which in turn produces a golden leaf phenotype. For example, transcriptional analysis has shown that the golden leaf phenotype in *Forsythia koreana* “Suwon Gold” may be associated with decreased *CHLH* expression (Zhang et al. 2022). The golden leaf phenotype in *Ginkgo biloba* mutants may be associated with altered expression of *HEMY*/*PPO* (protoporphyrinogen IX oxidase) and non-yellow coloring 1 (NYC1)/NYC1-like (NOL) (Li et al. 2018). In terms of Chl degradation, Mg-dechelatase, encoded by stay-green (SGR)/non-yellowing genes, is the initial enzyme that removes Mg^2+^ from Chl to produce pheophytin a (Pheo a) (Ren et al. 2007; Shimoda et al. 2016). Overexpression of *SGR* has been shown to induce a golden leaf phenotype in *Arabidopsis*, rice, and kiwifruit (*Actinidia chinensis*) (Park et al. 2007; Shimoda et al. 2016; Wu et al. 2024).

In this study, the primary difference between wild-type (WT) and JY *P. nigra* was leaf coloration (Fig. 1A). Pigment analysis showed that Chl a, Chl b, and Car levels were significantly lower in JY leaves compared with WT leaves. Moreover, the Chl a/b ratio was higher in JY leaves, whereas the total Chl/Car ratio was markedly lower than in WT leaves (Fig. 1B). RNA-seq analysis revealed clear clustering of three biological replicates from WT and JY leaves (Figure S1A). After removing redundant annotated transcripts, 120 differentially expressed genes (DEGs) were identified within the “chloroplast” Gene Ontology (GO) category (Figure S1B, C). These DEGs were significantly enriched in GO terms related to Chl metabolism, including “protoporphyrinogen IX biosynthetic process,” “Chl biosynthetic process,” and “Chl catabolic process” (Figure S1C). RNA-seq combined with RT-qPCR analysis showed that *PnSGR*, the initial gene in the Chl degradation pathway, was upregulated, whereas the downstream genes *PnPPH* and *PnPAO* were downregulated in JY leaves compared with WT leaves (Fig. 1C, D; Figure S2).

**Fig. 1:**
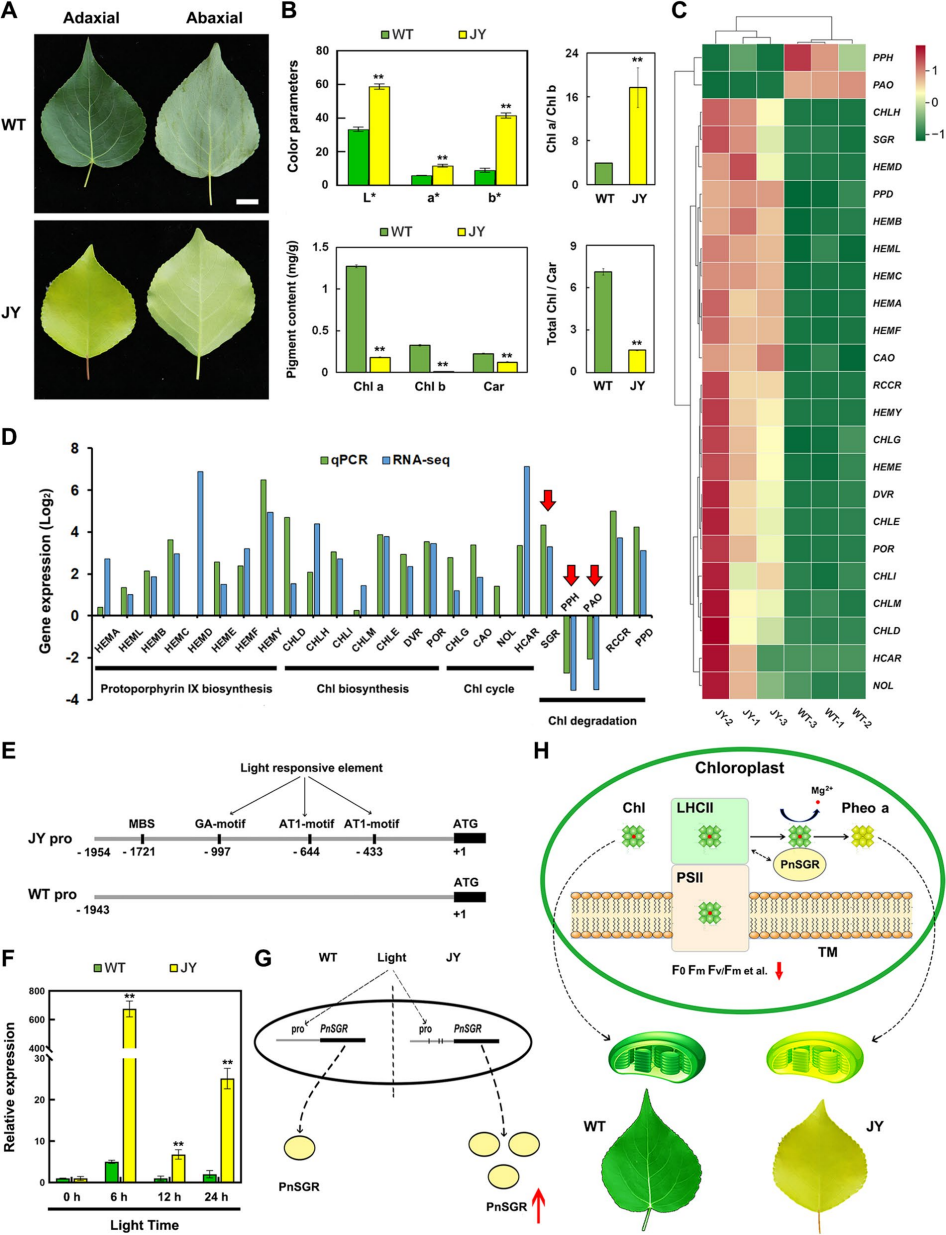
Working model of PnSGR in the formation of the JY phenotype in *Populus nigra*.** A** Leaf phenotype of WT and “Jinye” (JY) *plants with* leaf color parameters (L*,* a, b*) and pigment content. **B**, **C** Transcription levels of genes involved in Chl biosynthesis and catabolic pathways in WT and JY leaves. Red rectangles indicate upregulated genes, and green rectangles indicate downregulated genes. **D** Comparison between RT-qPCR and RNA-seq data. Red arrows highlight the upregulated *PnSGR* and the downregulated *PnPAO* in the Chl degradation pathway. **E** Differences in cis-acting elements between the *PnSGR* promoters of WT and “JY” plants. **F** Expression levels of *PnSGR* in WT and JY leaves under 0–24 h light treatment. **G** Three light-responsive cis-acting elements may promote *PnSGR* upregulation in JY plants. **H** PnSGR promotes the removal of Mg.^2+^ from Chl (mainly Chl a) to form Pheo a in chloroplasts. Chl is mainly derived from Chl–protein complexes (LHCII–PSII). Chl degradation results in leaf yellowing and alterations in PSII parameters, including F_0_, Fm, and Fv/Fm. PSII, photosystem II; LHCII, light-harvesting complex of PSII; TM, thylakoid membrane. Asterisks denote significant differences between WT and JY leaves (**p < 0.01; Student’s *t*-test). Error bars represent standard error (SE), *n* = 3

Sequence analysis revealed that the amino acid sequences of PnSGR were the same between WT and JY *P. nigra* plants (Figure S3). Transient expression assays showed that PnSGR–green fluorescent protein (PnSGR-GFP) fluorescence signals largely overlapped with chloroplast autofluorescence in tobacco leaf cells, confirming chloroplastic localization of PnSGR (Figure S4A). Furthermore, tobacco leaves transiently expressing PnSGR-GFP exhibited a yellowing phenotype compared with control leaves expressing GFP (Figure S4B). Pigment analysis demonstrated that Chl a, Chl b, and Car levels were significantly reduced in PnSGR-GFP expressing leaves, whereas Pheo a content was significantly increased relative to control leaves (Figure S4C, D). The PnSGR-GFP fusion gene was subsequently introduced into 84K poplar, generating transgenic seedlings (Figure S5). Three independent transgenic lines displayed distinct golden leaf phenotypes compared with WT poplar seedlings (Figure S6A). Pigment analysis revealed significantly lower Chl a, Chl b, and Car contents in transgenic leaves relative to WT leaves (Figure S6B), whereas Pheo a content was significantly increased (Figure S6C). In addition, the Chl a/b ratio was significantly higher and the total Chl/Car ratio significantly lower in transgenic leaves compared with WT leaves (Figure S6D, E). Expression of *PnPPD* and *PnRCCR* was also increased in JY leaves compared with WT leaves, although their effects on Chl degradation were less pronounced than those of *PnSGR* in tobacco transient expression assays (Figure S7). PnSGR functions as the initial and rate-limiting enzyme in Chl degradation. The results indicate that *PnSGR* overexpression accelerates Chl degradation, particularly of Chl a, leading to increased Pheo a accumulation and the golden leaf phenotype observed in tobacco and poplar. Moreover, the expression of nearly all Chl biosynthesis and metabolism genes was altered in transgenic poplar leaves compared with WT leaves (Figure S8).

Chl fluorescence parameters were compared between transgenic poplar and WT seedlings. F_0_, Fm, and Fv/Fm values were significantly lower in transgenic leaves than in WT leaves (Figure S9). Similarly, *PI*abs, *PI*tatol, and Δ*I*/*Io* values were also markedly reduced in transgenic seedlings (Figure S10A-D). OJIP curve analysis revealed that transgenic leaves exhibited significantly higher *V*j and *V*k values compared with WT leaves (Figure S10E-M). Transmission electron microscopy (TEM) further showed altered chloroplast ultrastructure in transgenic seedlings, characterized by widened gaps between thylakoid membranes (Figure S11). Collectively, these findings demonstrate that *PnSGR* overexpression impairs photosystem II (PSII) function and disrupts chloroplast structure, likely contributing to the reduced Chl content in transgenic poplar seedlings.

Sequence analysis showed that the *PnSGR* coding sequences were the same between WT and JY *P. nigra* (Figure S3); however, their expression levels differed (Fig. 1C), likely due to promoter variation. The ~ 2,000-bp promoter regions of *PnSGR* from WT and JY plants were isolated (Figure S12). Compared with the *PnSGR*(WT) promoter, the *PnSG*R(JY) promoter contained four unique cis-acting elements: one MYB binding site involved in drought-inducibility (MBS), one GA-motif, and two AT1-motifs (Fig. 1E; Figure S13). PlantCARE analysis classified the GA- and AT1-motifs as light-responsive elements. Consistent with this, *PnSGR* expression in JY leaves was markedly higher than in WT leaves under 6–24 h light exposure (Fig. 1F). Together, these findings indicate that the golden leaf phenotype of JY *P. nigra* is mainly attributable to reduced Chl content, driven by enhanced *PnSGR* expression, a key regulator of Chl degradation. Transient and stable expression analyses demonstrated that PnSGR localized to the chloroplasts and that its overexpression promoted Chl degradation, particularly of Chl a in the PSII–LHCII complex, to Pheo a (Fig. 1G, H). The increased expression of *PnSGR* in JY *P. nigra* appears to be related to light-responsive cis-acting elements in its promoter (Fig. 1G). These results provide new insights into the molecular basis of the golden leaf phenotype in JY *P. nigra* and suggest the potential to achieve golden leaf breeding in other ornamental plants through genetic engineering.

## Supplementary Information

Supplementary Material 1.

Supplementary Material 2.

Supplementary Material 3.

## Data Availability

All data are available in the manuscript.
