# Supplementary material for: Golden leaf formation of Populus nigra is associated with the up-regulation of Stay-Green expression
Source: Mol Hortic. 2026 Mar 6;6:22. doi: 10.1186/s43897-025-00204-9 (PMC12964669; doi:10.1186/s43897-025-00204-9)
Supplement: Supplementary file 1 — Supplementary Material 1. [file 43897_2025_204_MOESM1_ESM.pdf]

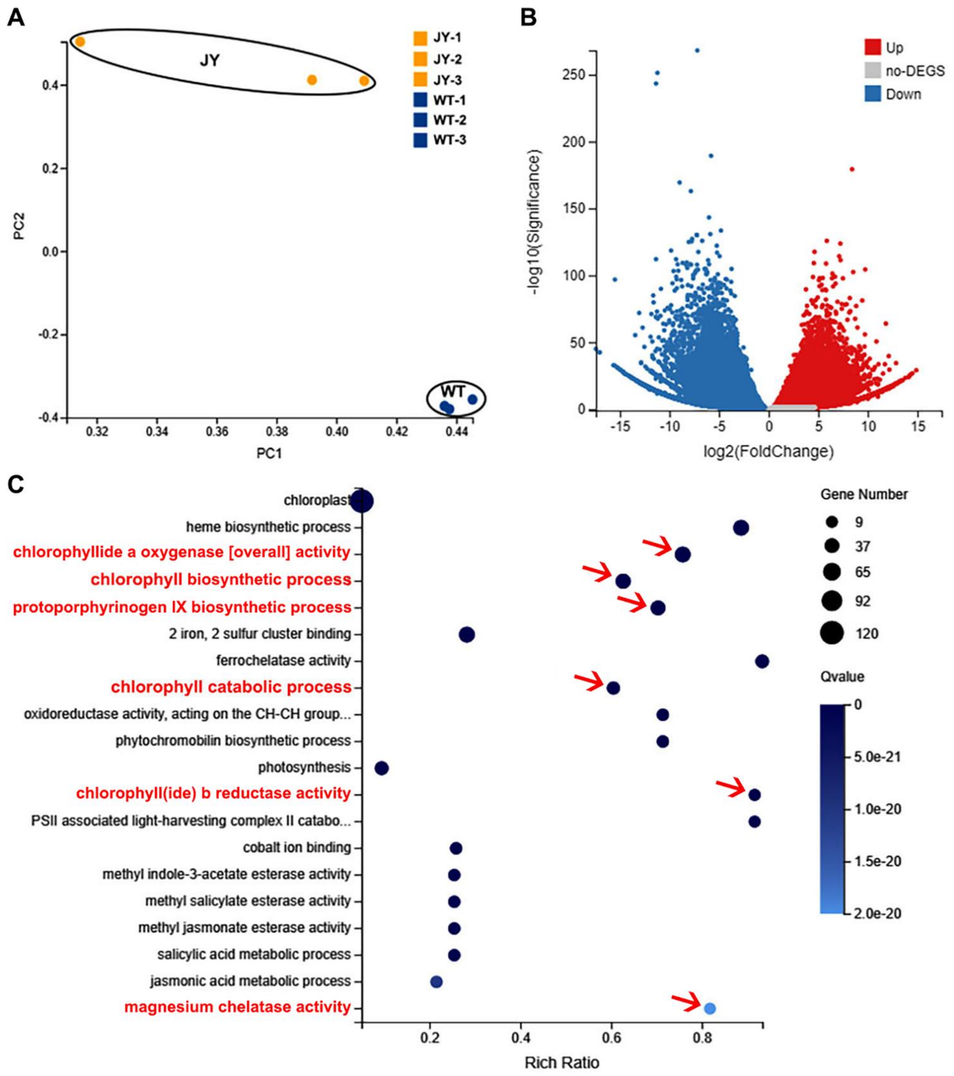

Figure S1. Identification of differentially expressed genes (DEGs) related to Chl biosynthesis and catabolism in the leaves of WT and JY *P. nigra* through RNA-seq. **A** Principal components analysis (PCA) of gene expression from six RNA-seq samples. **B** Volcano plots of the DEGs in WT versus JY leaves. **C** Gene Ontology (GO) enrichment analysis of the DEGs involved in the 'chloroplast' GO terms. The rich factor is the ratio of the number of DEGs annotated to a given GO pathway to the number of all genes annotated to the GO pathway. The Q-value is the corrected *p*-value and ranges from 0 to 1; a lower Q-value indicates greater intensity. Red arrows indicate the GO terms related to Chl metabolism.

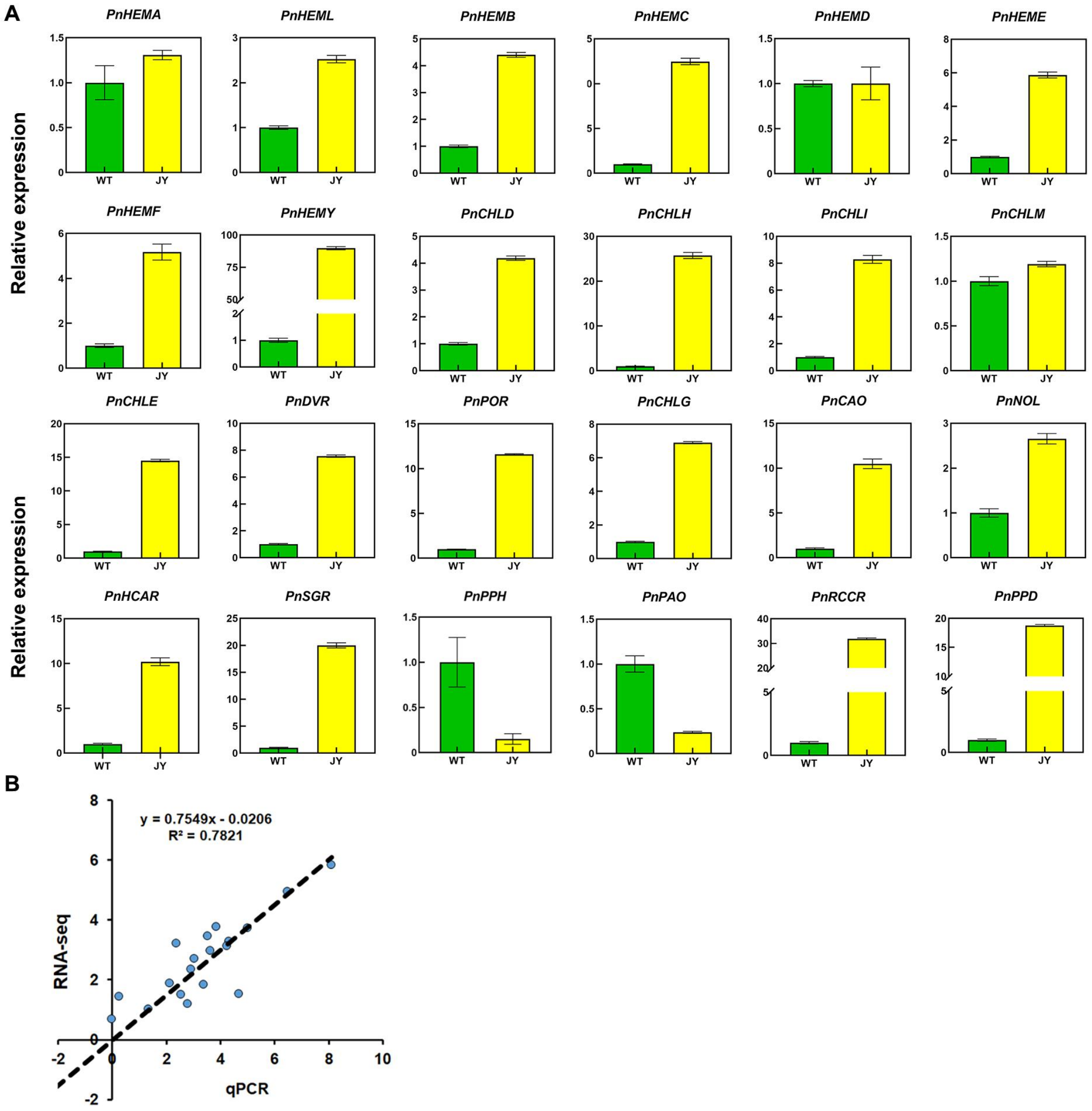

Figure S2. Real-time quantitative PCR (RT-qPCR) validation of genes involved in Chl biosynthesis and catabolic pathways. **A** The RT-qPCR of the relative expression of the genes involved in Chl biosynthesis and catabolic pathways, which are also divided into protoporphyrin IX biosynthesis, Chl biosynthesis, Chl cycle, and Chl degradation. **B** Correlation analyses between the RT-qPCR and RNA-seq data. Error bars show the SE (*n* = 3).

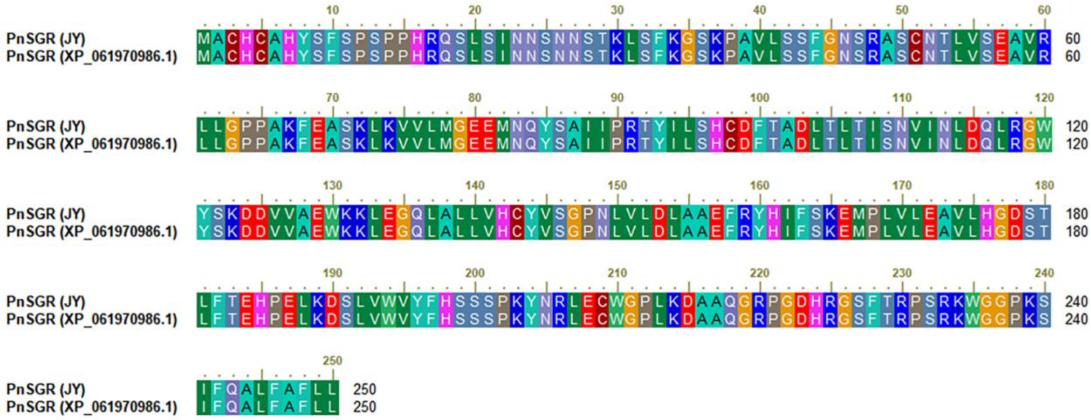

Figure S3. Amino acid sequence alignments of SGR proteins from wild-type (WT) and 'JinYe' (JY) *Populus nigra*.

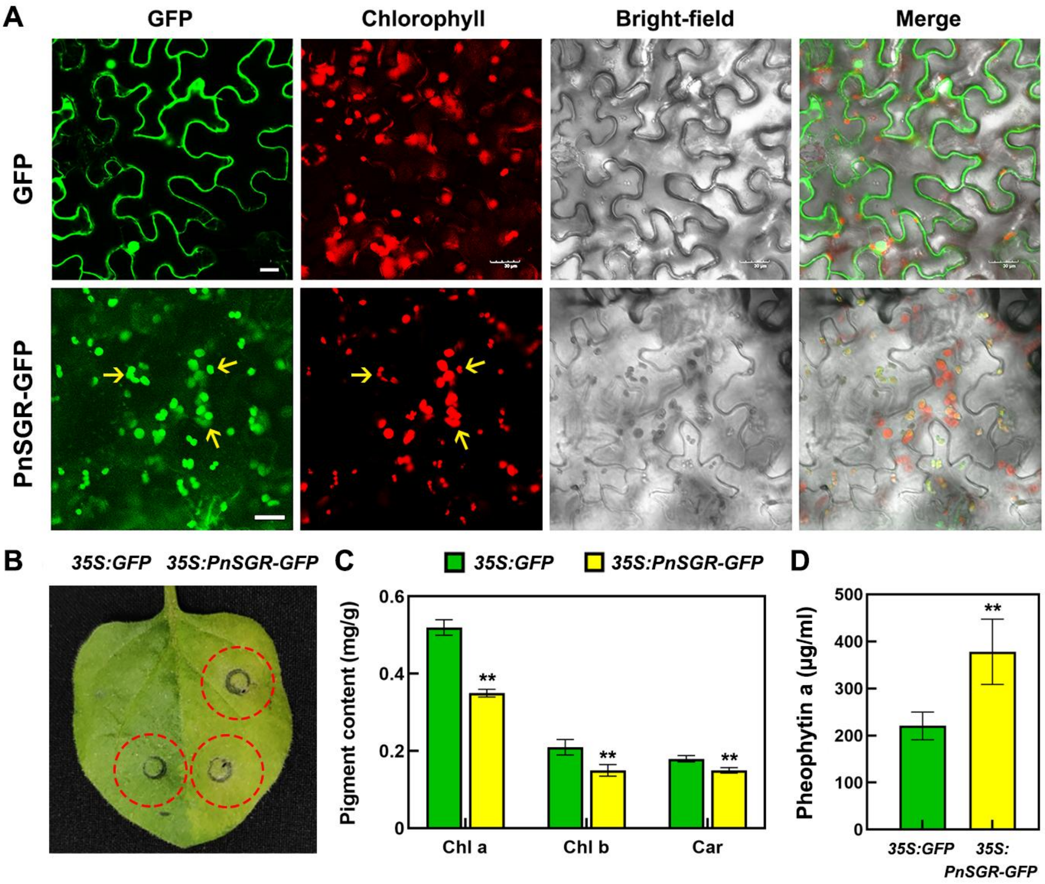

Figure S4. Subcellular localization of PnSGR and the effect of its transient expression on Chl content. **A** Confocal images of tobacco leaf cells transiently expressing GFP (control) and PnSGR-GFP. GFP fluorescence is green, and Chl spontaneous fluorescence is red. Yellow arrows indicate the overlap of PnSGR-GFP and Chl spontaneous fluorescence signals. **B** The leaf phenotype of the tobacco leaves transiently expressing GFP and PnSGR-GFP and their pigment content, including **C** Chl a, Chl b, carotenoid (Car), and **D** Pheophytin a (Pheo a). The infiltrated leaf areas are indicated by red circles. Asterisks indicate significant differences between WT and transgenic poplar (** p < 0.01; Student's t-test). Error bars represent SE (n = 6). Scale bars = 20 μm (a).

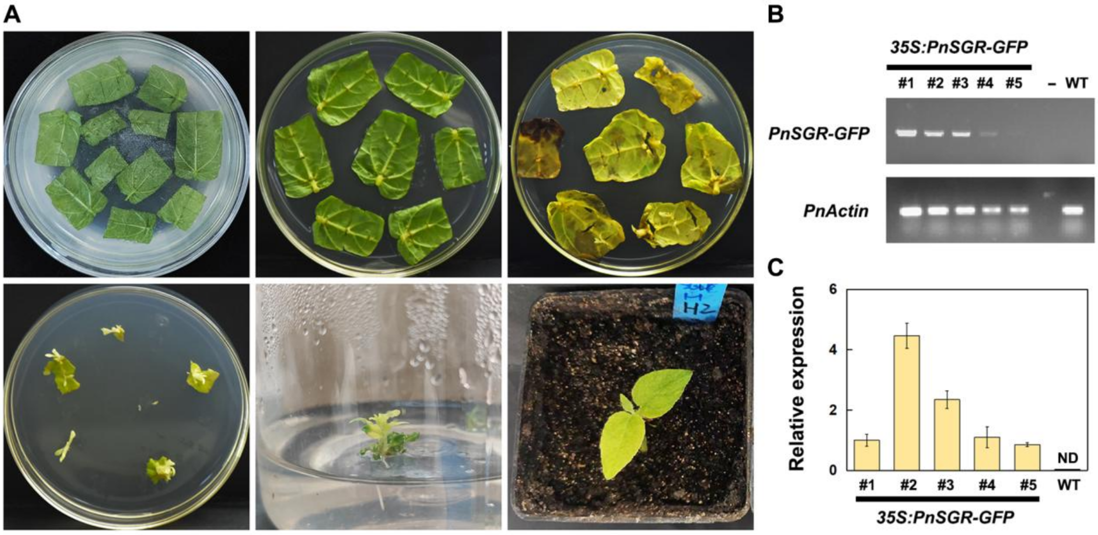

Figure S5. Genetic transformation and molecular characterization of *PnSGR-GFP* in poplar. **(a)** Genetic transformation and resistant plant screening in 84 K poplar with the *PnSGR-GFP* gene using the leaf plate method. Semi-quantitative **(b)** and real-time quantitative PCR **(c)** analysis of *PnSGR-GFP* transcription in the wild-type (WT) and transgenic poplar lines (#1–#5). ND: no data. Error bars show the standard error (SE) (n = 3).

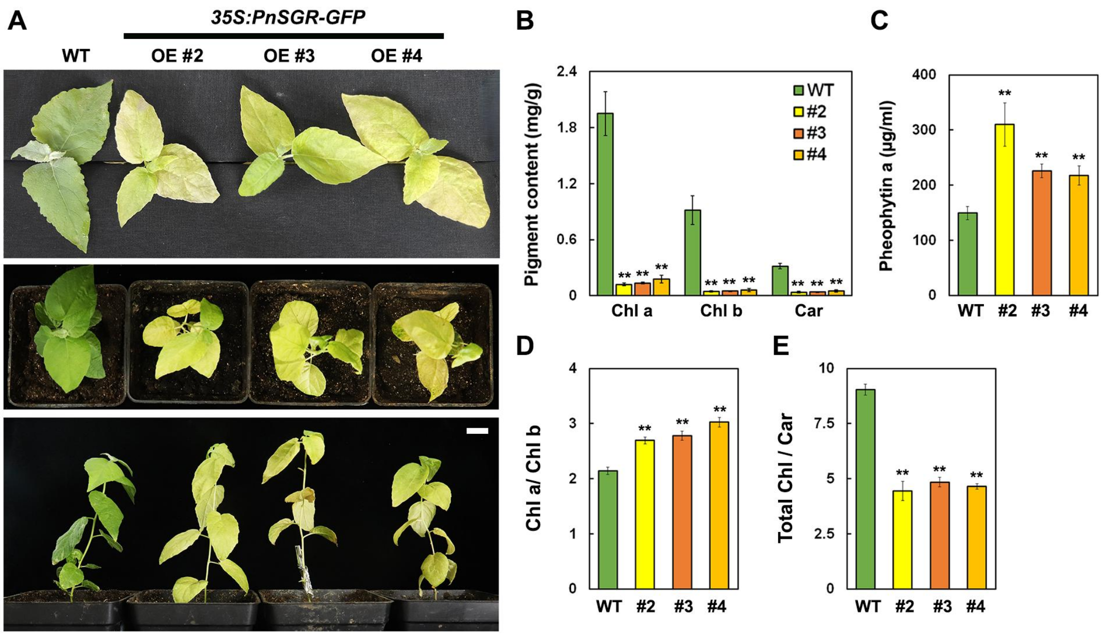

Figure S6. Leaf color phenotype and Chl content of WT and poplar seedlings overexpressing *PnSGR-GFP*. **A** Leaf phenotypes of WT and *PnSGR-GFP* transgenic poplar seedlings grown in the soil. **B** The Chl a, Chl b, Car, **C** and Pheo a content in the leaves of WT and *PnSGR-GFP* transgenic poplar and their ratios, **D** Chl a/b and **E** total Chl/Car. Asterisks indicate significant differences between WT and transgenic poplar (** $p < 0.01$; Student's *t*-test). Error bars represent SE ($n = 6$). Scale bars = 20 cm (a).

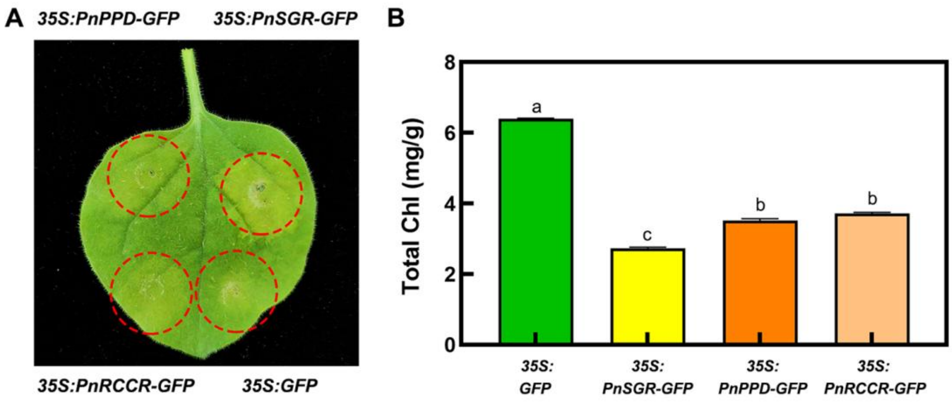

Figure S7. The leaf phenotype **(A)** of the tobacco leaves transiently expressing GFP, PnSGR-GFP, and PnPPD-GFP and PnRCCR-GFP and their total Chl content **(B)**. The infiltrated leaf areas are indicated by red circles. Different letters indicate significant differences (Student's t-test; $p < 0.01$). Error bars represent SE ($n = 6$).

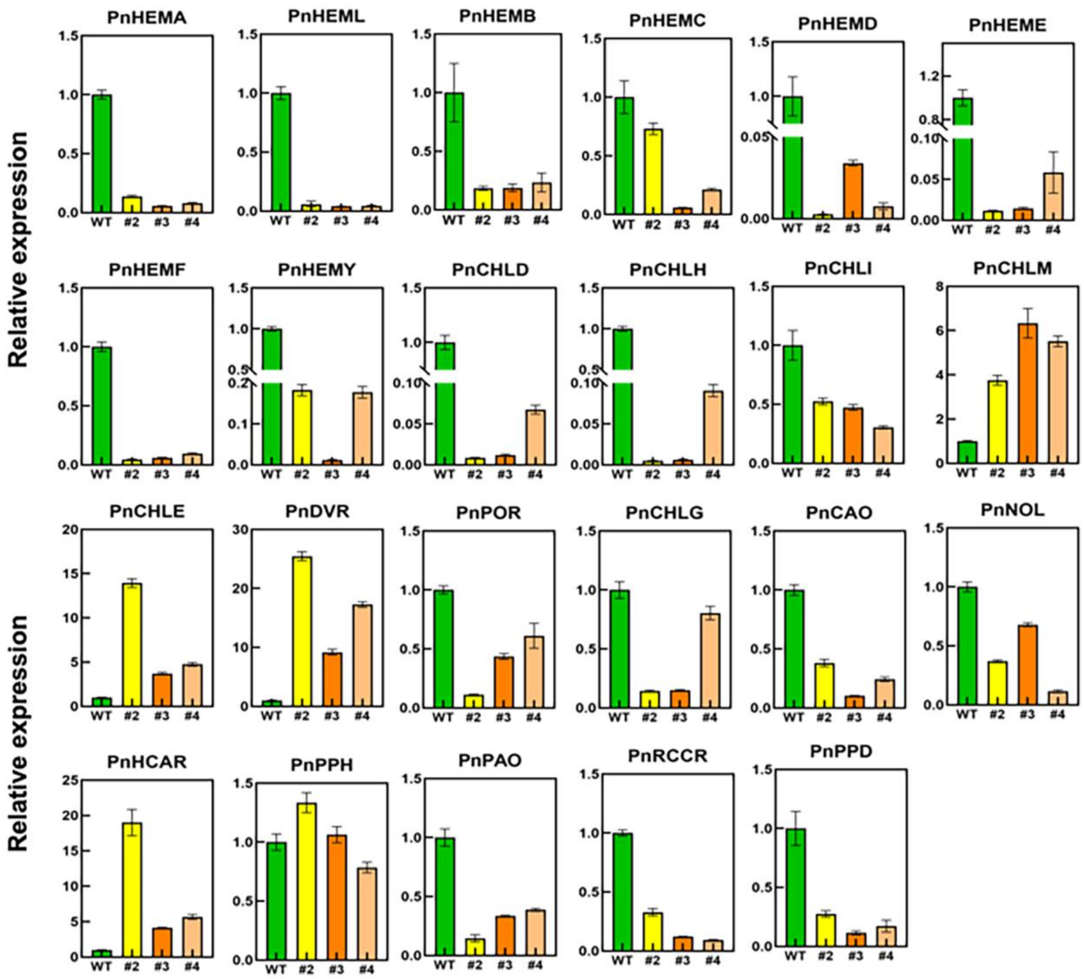

Figure S8. Real-time quantitative PCR (RT-qPCR) analysis of genes involved in Chl biosynthesis and catabolic pathways in the leaves of WT and *PnSGR-GFP* transgenic poplar seedlings. Error bars show the SE (n = 3).

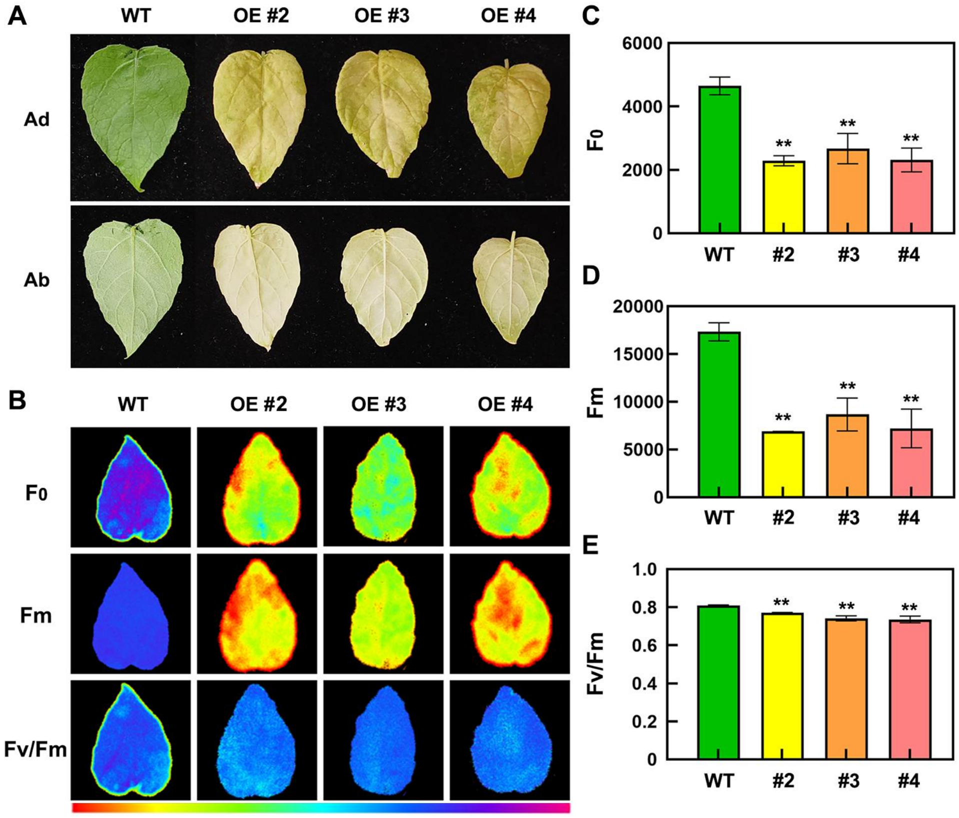

Figure S9. Chl fluorescence parameters in the leaves of WT and *PnSGR-GFP* transgenic poplar seedlings. **A** Leaf color of the adaxial and abaxial surfaces of WT and transgenic poplar seedlings, and their **B** Chl fluorescence images and values, including **C** F_0, **D** Fm, and **E** Fv/Fm. Asterisks indicate significant differences between WT and transgenic poplar (** $p < 0.01$; Student's *t*-test). Error bars represent SE ($n = 6$).

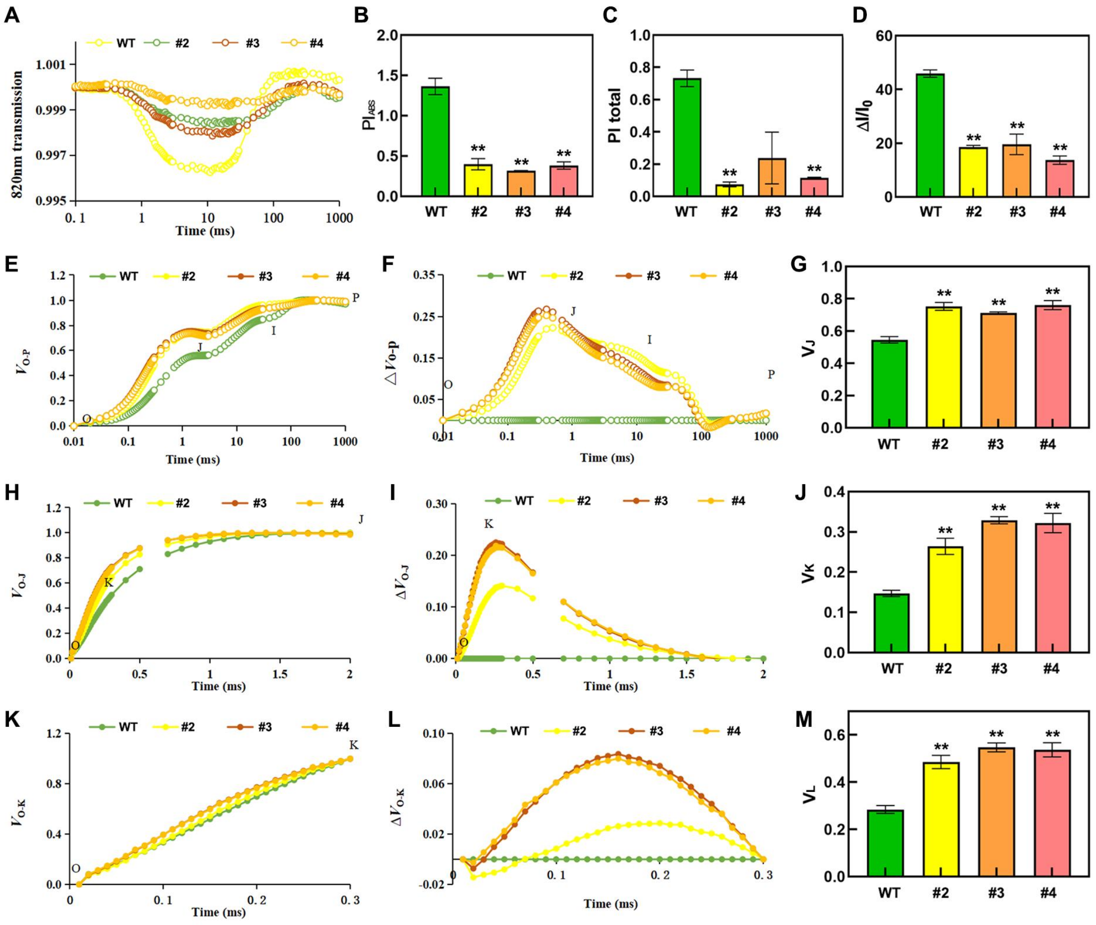

Figure S10. Chl fluorescence curves and parameters in the leaves of WT and *PnSGR-GFP* transgenic poplar seedlings. **A** The Chl fluorescence curves in the leaves of WT and transgenic poplar seedlings and the calculated parameters, including **B** the photosynthetic performance index (PI_{abs}) based on light absorption, **C** the total photosynthetic performance index (PI_{total}), **D** and the activity of PSI reaction center (△*I*/*Io*). On the OJIP curve, the corresponding time points when O, J, I, and P were 0.01, 2, 30, and 1000 ms, respectively. The $V_{O\text{-}P}$, $V_{O\text{-}J}$, and $V_{O\text{-}K}$ curves in the leaves of transgenic poplar seedlings were compared with those of the WT to obtain the **(E, F)** △$V_{O\text{-}J}$, **(H, I)** △$V_{O\text{-}K}$, and **(K, L)** △$V_{O\text{-}P}$ curves. The relative variable fluorescence of point J on the $V_{O\text{-}P}$ curve, point K on the $V_{O\text{-}J}$ curve, and point L on the $V_{O\text{-}K}$ curve are represented by **(G)** V_K, **(J)** V_L, and **(M)** V_J, respectively. Asterisks indicate significant differences between WT and transgenic poplar (** $p < 0.01$; Student's *t*-test). Error bars represent SE ($n = 6$).

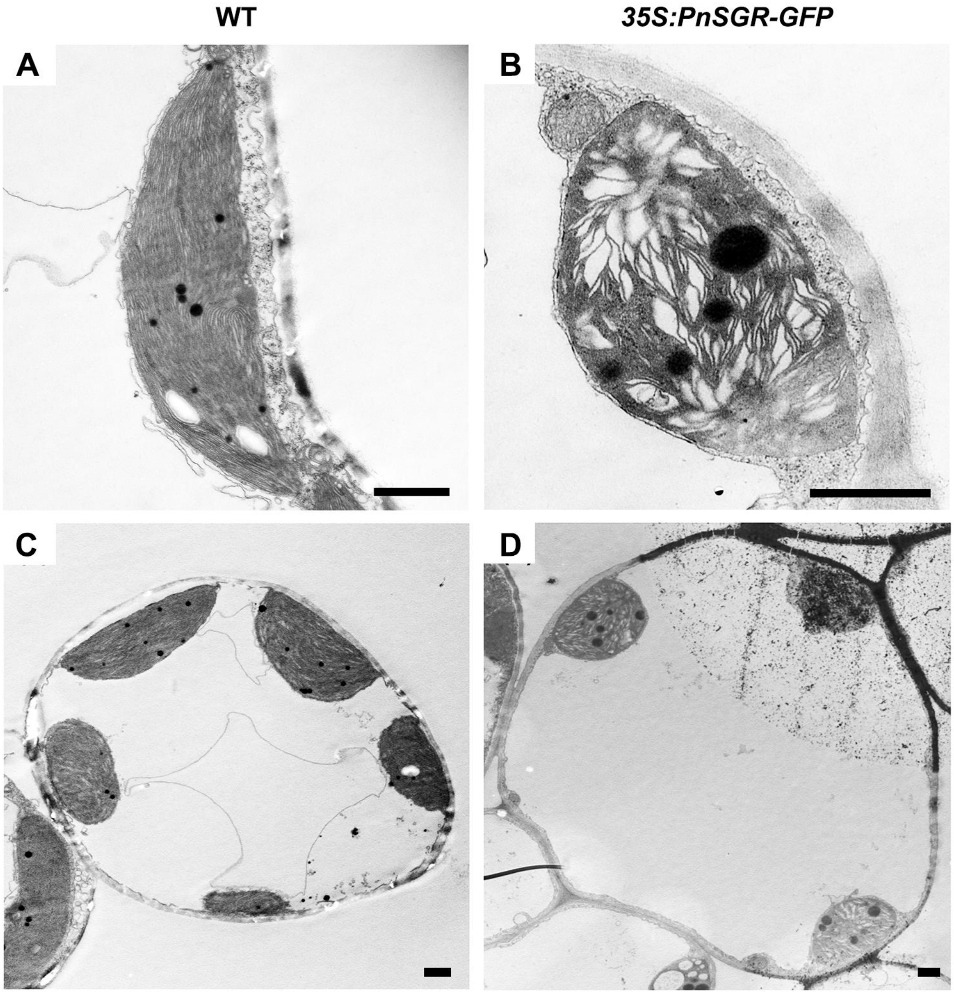

Figure S11. Ultrastructure of the chloroplasts of WT and *PnSGR-GFP* transgenic poplar seedlings. Transmission electron microscopy images of ultrathin sections of leaf cells from **(A, C)** WT and (**B, D**) *PnSGR-GFP* transgenic poplar. Scale bars = 200 nm.

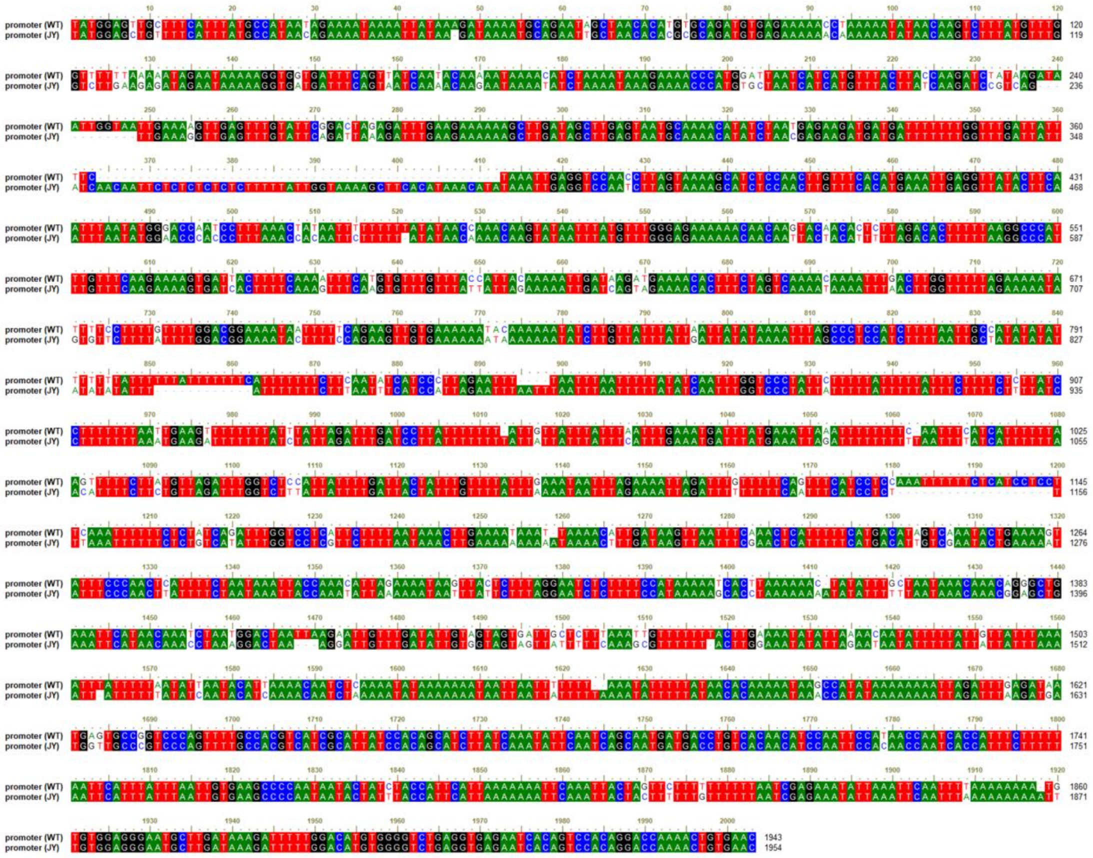

Figure S12. Alignment of promoter sequences of the *PnSGR* gene from wild type (WT) and 'JY' *Populus nigra*

promoter of *PnSGR* gene from 'JY' *Populus nigra* (-1954 bp)

TATGGAGCTGTTTTCATTTATGCCATAACAGAAAATAAAATTATAAGATAAAATGCAGAATTGCTAACAC
ACGCGCAGATGTGAGAAAAAACAAAAAATATAACAAGTCTTTATGTTTGGTCTTGAAGAGATAGAATA
AAAAGGTGATGATTTCAGTAATCAAAACAAGAATAAAATATCTAAAATAAAGAAAACCCATGTGCTAAT
CATCATGTTTACTTATCAAGATCCGTCAGTTGAAAGGTTGAGTTTGTATTCAGATTAAAGATTTGAAGA
MBS
AAAAAGCTTGATAGCTTGAGTAATGCAAAACATATCTAACGAGAAGATGATGATTTTTTTGGTTTGATT
ATTATCAACAATTCTCTCTCTCTCTTTTTATTGGTAAAAGCTTCACATAAACATATAAATTGAGGTCCAAT
CTTAGTAAAAGCATCTCCAACTTGTTTCACATGAAATTGAGGTTATACTTCAATTTAATATGGAACCCCAC
CCTTTAAACCACAATTCTTTTTATATAACAAAACAAGTATAATTTATGTTTGGGAGAAAAAACAACAATT
ACTACATTTTTAGACACTTTTTAAGGCCCATTTGTTTCAAGAAAAGTGATCACTTTTCAAAGTTTCAAG
TGTTTGTTTATTATTAGAAAAATTGATCAGTAGAAAACACTTTCTAGTCAAAATAAAATTTAACTTGGTT
TTTAGAAAAATAGTGTTCTTTTATTTTGGACGGAAAATACTTTTCCAGAAGTTGTGAAAAAAAATAAAAA
AATATCTTGTTATTTATTGATTATATAAAATTTAGCCCTCCATCTTTTAATTGCTATATATATATATATATTTAT
TTTTTTCTTTAATTTCATCCATTAGAATTTAATTTAATTTAATTTTTATATCAATTTGGTCCCTATTATTTTTA
TTTTTATTTCTTTTCTTTTATCCTTTTTTTAAATGAAGATTTTTTTATCTATTAGATTTGATCCTTATTTTTTT
GA-motif
TATTATTATTTATTTCATTTGAAATGATTTATGAAATTAGATTTTTTTTTTAATTTTATCATTTTTTAACATTT
TCTTCTGTTAGATTTGGTCTTTATTATTTTGATTACTATTTGTTTTATTTAAAATAATTTAGAAAATTAGATT
TTTTTTTCAATTTCATCCTCTTTTAAATTTTTTCTCTGTCATATTTGGTCCTCGTTCTTTTAATAAACTTGA
AAAAAAAAATAAAACTTTGATAAGTTAATTTCGAACTCATTTTTCATGACATTGTCGAATACTGAAAAA
TATTTCCCAACTTATTTTCTAATAAATTACCAAATATTAAAAAAATAATTTATTCTTTAGGAATCTCTTTTCC
AT1-motif
ATAAAAAGCACCTAAAAAAAAATATATTTTTTAATAAACAAACGGAGCTGAAATTCATAACAAACCTAAA
GGACTAAAGGATTGTTTGATATTGTGGTAGTAGTTATTTTTCAAAGCGTTTTTTACTTGGAAATATATTAG
AATAATATTTTTATTATTATTTAAAATTATTTTTTATATCAATACATCAAAACAATCTAAAAATATAAAAAA
AT1-motif
ATAATTAATTATTTTTAAAATATTTTTATAACACAAAAATAAACCATATAAAAAAAATTAGATTTAAGATG
ATGGTTGCCCGTCCCAGTTTTGCCACGTCATCGCATTATCCACAGCATCTTATCAAATATTCAATCAGCA
ATGATGACCTGTCACAACATCCAATTCCACAACCAATCACCATTTCTTTTTAATTCATTTATTTAATTGTG
AAGCCCCAATAATACTATTTACCATTCATTAAAAAAATTCAAATTACTACTTTTTTGTTTTTTAATCGAGA
AATATTAAATTCAATTTAAAAAAAAAAATTTGTGGAGGGAATGCTTGATAAAGATTTTTGGACATGTGGG
GTCTGAGGTGAGAATCACAGTCCACAGGACCAAAACTGTGAAC +1

Figure S13. The cis-acting elements in the promoter of the *PnSGR* gene from 'JY' *Populus nigra* distinct from that in wild type (WT)
