# Supplementary material for: Golden leaf formation of Populus nigra is associated with the up-regulation of Stay-Green expression
Source: Mol Hortic. 2026 Mar 6;6:22. doi: 10.1186/s43897-025-00204-9 (PMC12964669; doi:10.1186/s43897-025-00204-9)
Supplement: Supplementary file 3 — Supplementary Material 3. [file 43897_2025_204_MOESM3_ESM.docx]

**Golden leaf formation of** ***Populus nigra* is associated with the up-regulation of *Stay-Green* expression**

**Wanting Fu ^a,1^, Zimeng Li ^a,1^, Xiaoou Zhai ^b^, Haizhen Zhang ^a^, Shuang Feng ^c, *^, Aimin Zhou ^a, *^**

^a^ College of Horticulture and Landscape Architecture, Northeast Agricultural University, Harbin 150030, China

^b^ Heilongjiang Forest Botanical Garden, Harbin 150046, China

^c^ Large-Scale Instrument and Equipment Sharing Service Platform, Northeast Agricultural University, Harbin 150030, China

^1^ Authors contributed equally to the work.

* Corresponding authors, E-mail: shuangfeng@neau.edu.cn; aiminzhou@neau.edu.cn

**Materials and methods**

**Plant materials**

Wild-type (WT) *Populus nigra* and golden leaf variety *P. nigra* cv. 'Jinye' (JY) materials were grown in the nursery of Northeast Agricultural University (Harbin, China). Cuttings and seedlings were grown in a greenhouse under a 12-h light/12-h dark photoperiod (150 μmol m^−2^ s^−1^ light) at 26 °C.

**Measurement of the pigment content**

The leaf color parameters L*, a* and b* were measured using a Chroma meter (CR-400, Konica Minolta Sensing, Japan). Pigments were extracted from the leaf tissues using 80 % ice-cold acetone. Quantification of Chl a, Chl b, and carotenoids (Car) was performed using a UV/Vis spectrophotometer at light absorption values of 470 nm, 645 nm, and 663 nm, respectively. The measurements were conducted in darkness to avoid degradation of photosynthetic pigments. The measurements were performed with six biological replicates and three technical replicates. The Pheo a content was measured using plant Pheophytin a ELISA Kit (Shanghai Enzyme-linked Biotechnology Co., Ltd., China) according to the manufacturer’s protocol.

**RNA sequencing (RNA-seq) and differentially expressed genes (DEGs) analysis**

Total RNA was extracted from the leaves of *P. nigra* plants using TRIzol reagent (Invitrogen, Carlsbad, CA, USA). RNA-seq and data processing were performed as described previously (Zhou et al. 2017). The expression levels of unigenes were calculated according to fragments per kilobase of transcript per million mapped reads (FPKM). Genes with false discovery rate (FDR) ≤ 0.05 and log2 ratio ≥ 1 were considered as DEGs. The DEGs were mapped against the gene ontology (GO) databases, and significant pathways were identified at *p* ≤ 0.05.

**Real-time quantitative PCR (****RT-qPCR)**

Total RNA was extracted from the leaves using RNAiso Plus Kit (TaKaRa, Shiga, Japan) and reverse-transcribed with the PrimeScript RT reagent kit with gDNA Eraser (TaKaRa). RT-qPCR was performed with the SYBR Premix Ex Taq kit (TaKaRa) using the CFX96 real-time PCR detection system (Bio-Rad, Hercules, CA, USA). *PtActin* was used as the internal reference gene. Three biological and three technical replicates were performed per sample. The primers are listed in Supplementary Material 2.

**Subcellular localization**

For subcellular localization, the open reading frame (ORF) (without the stop codon) of *PnSGR* gene was amplified through PCR and cloned into the pCAMBIA1300-GFP vector. The construct was transformed into the *Agrobacterium tumefaciens* strain EHA105 and then cotransformed into tobacco (*Nicotiana benthamiana*) using *Agrobacterium*-mediated tobacco leaf infection. The tobacco leaf epidermis was visualized using confocal laser scanning microscopy (CLSM; Olympus FV3000, Tokyo, Japan). GFP signals were detected using 500–530 nm emission filters. Chl autofluorescence signals were detected using a 620–680 nm emission filter. The primers used are listed in Supplementary Material 2.

**Genetic transformation of the 84K (P. alba × P. glandulosa)** **poplar**

The pCAMBIA1300-PnSGR-GFP construct was transformed into 84 K poplar using the *Agrobacterium*-mediated leaf discs method as previously described (He et al. 2018; Hu et al. 2022; Yao et al. 2016). Transgenic plants were identified by reverse transcription PCR (RT-PCR) and RT-qPCR. *PtActin* was used as the internal reference gene.

**Transmission electron microscopy (TEM)**

Ultrathin (60–80 nm) sections of Arabidopsis leaves were prepared as previously described (Zhou et al. 2016). Sections were poststained with uranyl acetate and lead citrate and observed under a H-7500 transmission electron microscope (Hitachi, Tokyo, Japan) operated at 80 kV.

**Chl fluorescence parameters**

The fully expanded leaves of poplar plants were measured and dark-treated for 30 min before measurement. The minimum Chl fluorescence (F_0_), maximum Chl fluorescence (Fm), and maximum quantum yield of PSII photochemistry (Fv/Fm) were measured using kinetic chlorophyll fluorescence imaging systems (FluorCam, PSI, Czech Republic). The Chl fluorescence induced dynamic (OJIP) curve and 820 nm light reflection curve (*MR*820) in the leaves were measured using a Multi-Function Plant Efficiency Analyser (M-PEA; Hansatech Ltd., King's Lynn, UK) as previously described by Wang et al. (2022). The OJIP curve was analyzed using a JIP test using the method of Strasser et al. (1995). The photosynthetic performance index (*PI*_ABS_) and the total photosynthetic performance index (*PI*_total_) were obtained. The Δ*I*/*Io* reflected the activity of the PSI reaction center, and Δ*I*/*Io* was considered the difference between the maximum and minimum values of the *MR*820 curve. The measurements were performed with six biological replicates and three technical replicates.

**Cloning and analysis of the promoters**

The cloning of the promoter was performed using specific primers with DNA as a template. The primers used are listed in Supplementary Material 2. Cis-acting elements in the promoters were analyzed using the PlantCARE program (http://bioinformatics.psb.ugent.be/webtools/plantcare/html/).

**Gene and accession numbers**

The RNA-seq sequence data were submitted to the National Center for Biotechnology Information (NCBI) Sequence Read Archive (SRA) database under the accession number PRJNA1204378. *PnSGR* accession: XP_061970986.1.

**Statistical analyses**

Data obtained were analyzed using one-way analysis of variance in SPSS software (SPSS, Inc., Chicago, IL, USA), and statistically significant differences were calculated using Student’s t-test, with *p* < 0.01 set as significance thresholds.

**Abbreviations**

Chl chlorophyll

Car carotenoids

LHC light-harvesting Chl-binding complexes

PS Photosystems

GluTR glutamyl-tRNA reductase

ALA 5-aminolevulinic acid

GSAT glutamate 1-semialdehyde aminotransferase

MgCh Mg-chelatase

Mg-ProtoME Mg-Proto monomethylester

Chlide chlorophyllide

SGR stay-green

Pheo a Pheophytin a

CHLG Chl synthase

CAO Chlide a oxygenase

HCAR 7-hydroxymethyl Chl a reductase

PPH pheophytin pheophorbide hydrolase

PAO pheophorbide a oxygenase

**Reference**

He F, Wang HL, Li HG, Su Y, Li S, Yang Y, Feng CH, Yin W, Xia X. PeCHYR1 a ubiquitin E3 ligase from *Populus euphratica* enhances drought tolerance via ABA-induced stomatal closure by ROS production in *Populus*. Plant Biotechnol J. 2018; 16: 1514–1528.

Hu X, Wang S, Zhang H, Zhang H, Feng S, Qiao K, Lv F, Gong S, Zhou A. Plant cadmium resistance 6 from *Salix linearistipularis* (*SlPCR6*) affects cadmium and copper uptake in roots of transgenic *Populus*. Ecotoxicol Environ Saf. 2022; 245: 114116.

Wang Y, Yu Y, Zhang H, Huo Y, Liu X, Che Y, Wang J, Sun G, Zhang H. The phytotoxicity of exposure to two polybrominated diphenyl ethers (BDE47 and BDE209) on photosynthesis and the response of the hormone signaling and ROS scavenging system in tobacco leaves. J Hazard Mater. 2022; 426: 128012.

Yao W, Wang S, Zhou B, Jiang T. Transgenic poplar overexpressing the endogenous transcription factor ERF76 gene improves salinity tolerance. Tree Physiol. 2016; 36: 896–908.

Zhou A, Ma H, Liu E, Jiang T, Feng S, Gong S, Wang J.Transcriptome sequencing of *Dianthus spiculifolius* and analysis of the genes involved in responses to combined cold and drought stress. Int J Mol Sci. 2017; 18: 849.
